# Wild Ungulates and Cattle Have Different Effects on Litter Decomposition as Revealed by Fecal Addition in a Northeast Asian Temperate Forest

**DOI:** 10.1002/ece3.70529

**Published:** 2024-11-14

**Authors:** Yongchun Hu, Jiawei Feng, Hongfang Wang, Jianping Ge, Tianming Wang

**Affiliations:** ^1^ Ministry of Education Key Laboratory for Biodiversity Science and Engineering, College of Life Sciences Beijing Normal University Beijing China; ^2^ National Forestry and Grassland Administration Key Laboratory for Conservation Ecology of Northeast Tiger and Leopard Beijing China

**Keywords:** litter decomposition, livestock, nutrient dynamics, temperate forest ecosystem, ungulate

## Abstract

Litter decomposition is critical for maintaining productivity and nutrient cycling in forest ecosystems. Large herbivores play an essential role in determining the processes of nutrient cycling. Asian temperate forests are becoming degraded and fragmented by the widespread intensification of anthropogenic activities, including excessive livestock grazing. However, the effects of livestock grazing and wild ungulates on forest litter decomposition remain less explored. In this study, we used a litterbag experiment to investigate the effects of the addition of cattle (*Bos taurus*) and sika deer (*Cervus nippon*) feces on litter decomposition. The study was conducted in Northeast China from July 2022 to October 2023. We found that the addition of deer feces significantly reduced litter decomposition, but the addition of cattle feces greatly increased litter decomposition. The presence of cattle and deer excrement significantly accelerated the release of C after 1 year of litter decomposition. Compared with the results of the control group (no addition of feces), the addition of cattle and sika deer feces increased C release by 37.45% and 22.69%, respectively. Fecal addition increased the release of N; however, for the three treatment groups, the maximum accumulation of N occurred in the middle of litter decomposition, which may have been due to the initial chemical quality of the leaves and snow melt as well as nutrient limitations at the sites. Compared with the results of the control group, P release in the feces of cattle increased by 4.35%, but P release in the feces of deer decreased by 27.55%. This work highlights that feces deposition by large herbivores (e.g., wild or domestic) in the forest has nonequivalent effects on litter decomposition. Such effects may further alter the nutrient cycling in temperate forest ecosystems, with far‐reaching effects on the ecosystem that deserve closer attention. We suggest that conservation managers should seek evidence‐based interventions to optimize livestock use of forest habitats shared with wildlife.

## Graphical abstract

Here we used a litterbag experiment to investigate the effects of the addition of cattle and sika deer (Cervus nippon) feces on litter decomposition in Northeast China. Our work highlights that feces deposition by large herbivores (e.g., wild or domestic) in the forest have nonequivalent effects on litter decomposition. Such effects may further alter the nutrient cycling in temperate forest ecosystems, with far‐reaching effects on the ecosystem that deserve closer attention.

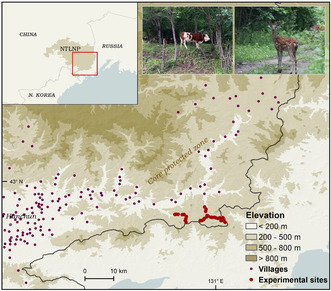

## 1 Introduction

Terrestrial plants globally produce 122 Pg of organic carbon (C) per year through photosynthesis (Beer et al. 2010). About half of the organic C is converted to dead organic matter (“litter”) (Cai et al. 2021; Cebrian 1999). Litter decomposition is essential for carbon and nutrient cycling globally and releases various elements from plants and returns them to the soil, providing a source for plant roots to absorb nutrients and facilitating the exchange of chemical elements within the ecosystems (Chollet et al. 2021; Wang et al. 2021; Zhang et al. 2022). It is estimated that over 60% of the mineral components and over 90% of the nitrogen and phosphorus in plant nutrition are recycled from nutrients returned to the soil by plants (Cornwell et al. 2008). Forest litter is a metabolic product produced by forest plants as they grow and develop (Prescott and Vesterdal 2021). The nutrient dynamics of various decomposition stages contribute significantly to the nutrient sustainability of terrestrial ecosystems (Naeem et al. 2022), soil organic matter accumulation (Zhang et al. 2022), and nutrient balance (Sun et al. 2018). The different rates of decomposition and mineralization of litter greatly affect the supply rates of the main nutrients required for forest growth (Fukasawa et al. 2014); litter decomposition provides the majority of the energy required to regulate biogeochemical processes controlled by saprophytic organisms (Du et al. 2020; Veen et al. 2019). Organic matter decomposition is dependent on the local climate, the activity of coprophagous invertebrates and soil microorganisms (Banegas et al. 2015), and the chemical composition of organic matter (Parton et al. 2007; Sun et al. 2018). In balanced native ecosystems, organic matter decomposition is synchronized with plant growth and development, as well as effective utilization of C and other nutrients (Moore et al. 2006); however, anthropogenic disturbances may retard or accelerate decomposition by altering the functional compartments of the ecosystem (Banegas et al. 2015; Yang and Wang 2019).

Large herbivores, both wild and domestic, are keystone species in many forest ecosystems and have long‐term and large‐scale influences on ecological functioning (Kastovská, Mastny, and Konvicka 2024; Öllerer et al. 2019). Large herbivores have important effects on litter decomposition through three common mechanisms: consumption of plant material, trampling of soil and surface vegetation, and defecation (Jiang et al. 2024; Pastor et al. 1993; Swain, Leroux, and Buchkowski 2023). They may alter the feedback between plant communities and the decomposer subsystem (Bobbink, Hornung, and Roelofs 1998). The modification of plant community composition as a consequence of herbivory could alter the quantity and quality of plant litter materials that are provided to the decomposer subsystem, which in turn affects litter decomposition and belowground nutrient cycling (Kasahara et al. 2016). For example, Chollet et al. (2021) reported that the preference of deer for plants may change litter decomposability; specifically, deer promote the growth of unpalatable species by preferentially eating the most palatable species. Domestic cattle (*Bos taurus*), which is direct descendant of aurochs (*B. primigenius*), feed extensively and act as herbivores to filter out plant species with high nutritional value and greater palatability within the plant community (Öllerer et al. 2019). Overall, the expected reductions in litter quantity and quality should slow decomposition processes and nutrient cycling (Bardgett and Wardle 2003). Large herbivores also excrete urine and feces, a source of organic matter that is more easily decomposable than recalcitrant plant litter is (Wei et al. 2021). Waste deposition by large herbivores may accelerate litter decomposition (Bardgett and Wardle 2003). Trampling can fragment plant material, which then promotes the incorporation of litter into soil (Mancilla‐Leytón, Sánchez‐Lineros, and Vicente 2013). This, in turn, enhances litter decomposition by facilitating microbial activity across various environments (Wei et al. 2021). Livestock and wild ungulates have different body weights, hoof pressures and movement patterns, which affect litter breakage and decomposition rates (Öllerer et al. 2019).

The temperate mixed forest in Northeast Asia represents one of the key regional priorities for biodiversity‐carbon synergy protection in Asia, supporting critical populations of two large cats, the endangered Amur tiger (*Panthera tigris altaica*) and the critically endangered Amur leopard (*P. pardus orientalis*) (Feng et al. 2021; Wang et al. 2016). To improve the forest conservation outlook, the commercial logging of natural forests has been halted since 1998 in Northeast China (Fu et al. 2022; Yang et al. 2013). As an alternative to logging, cattle grazing within forests has become a prevalent land use and is the main cause of forest degradation and biodiversity decline in Northeast China (Wang et al. 2019, 2016). In seeking to create a protected area for tigers and leopards, the Chinese government recently established a large national park (*ca*. 14,000 km^2^) along the China‐Russia border. The management department intends to shift forest management away from livestock grazing to restore habitat for dwindling populations of tigers and leopards, while simultaneously providing important ecological services to support human livelihoods (Feng et al. 2021). However, cattle, as an integral part of the temperate forest ecosystem, are vital for the livelihoods of locals and the economies of the region. If forest grazing, which has continued for decades, was suddenly discontinued in this area, communities would suffer greatly, increasing degree of the conflict between local people and nature conservation. Thus, applying multiple indices to achieve comprehensive quantification of grazing impacts is essential if protected areas are to balance the need to use nature and to ensure that wild animals can survive and reproduce. Litter decomposition and livestock excreta are two important sources of nutrient replenishment in forest soils. Litter decomposition, as a key link in biogeochemical cycling, is a crucial pathway for aboveground plant residues to return organic matter and nutrients to the soil, thereby preserving soil fertility in terrestrial ecosystems and playing an important role in maintaining ecosystem structure and function. Livestock and wild ungulates have varied effects on litter decomposition due to differences in their physiology, diet, behavior, numbers and management (Bernes et al. 2018; Walker, Anderson, and Fugal 2015). Cattle, for example, weigh more than 400 kg, exceeding the body size of most wild ungulates in Northeast China, which ranges from 30 to 110 kg (Dou et al. 2019). The effects of large herbivores grazing on ecosystems can be strongly context dependent (e.g., forest type, wildlife type, stocking levels and season) (Pringle et al. 2023). Properly managed and targeted livestock grazing can benefit both forest ecosystem functions and native biodiversity by increasing habitat diversity and quality (Giorgis et al. 2020; Öllerer et al. 2019). To date, few studies have investigated the effects of large wild ungulates and livestock excrement on litter decomposition in temperate forest ecosystems. Wild sika deer and domesticated cattle share the same trophic level; therefore, determining their impact on litter decomposition and nutrient cycling in a Northeast Asian temperate forest is critical.

In this study, we conducted a field litter decomposition experiment in Northeast China through the addition of cattle and sika deer feces to forest litter. We aimed to determine the litter decomposition process as well as the dynamic patterns of C, N, and P over the course of 415 days. Here, litter mass loss, the decomposition rate (*k* value), and C, N, and P release were used as indicator variables to evaluate the litter decomposition speed and nutrient dynamics. Specifically, we addressed the following queries: (1) What is the difference in litter decomposition between the treatments with cattle feces and those with sika deer feces? (2) Is the release pattern of nutrients in the litter decomposition process consistent among the treatments with cattle feces and sika deer feces added? Our study aims to provide practical restoration recommendations to best restore the biogeochemical functions of the ecosystems.

## 2 Materials and Methods

### 2.1 Study Area

**FIGURE 1:**
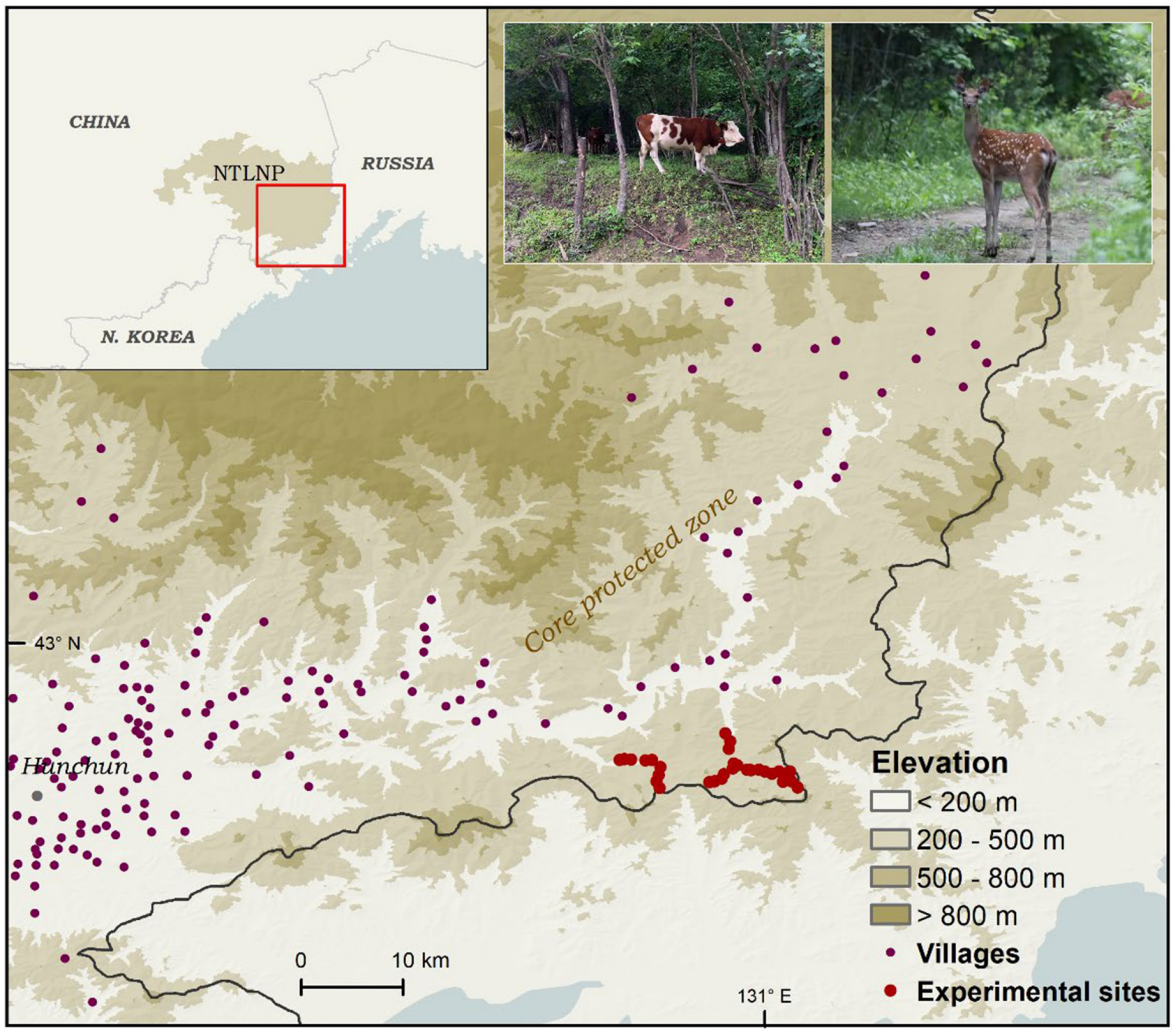
Map of experimental sites (dark red solid circles) in core protected zone of Northeast Tiger and Leopard National Park (NTLNP), with the insets showing the photographs of sika deer (*Cervus nippon*) and cattle (*Bos taurus*).

The study was conducted in the eastern part of Northeast Tiger and Leopard National Park (NTLNP) along the China‐Russia border (Figure 1). The study area is the core protected zones of the park and has tracts of extensive continuous forest. The elevation ranges from 5 to 1477 m. The region experiences a frost‐free period of 110 to 160 days per year with an average annual temperature of 5.60°C (±1.30°C) due to its temperate continental monsoon climate. From 1990 to 2010, there was an average of 618 mm (±68 mm) of precipitation annually, with the majority falling between June and August (Wang et al. 2018). The park was established after farmers started to settling there, just as many other natural reserves in China. More than 62,300 people live in 130 settlements inside the park, and their main sources of income are agriculture, gathering of nontimber forest products, and livestock husbandry. Livestock are essential for the rural development of the region, with an average density of 8–11 cattle per km^2^ and a maximum density of 30–50 cattle per km^2^ in grazed forests (Feng et al. 2021).

### 2.2 Experimental Design

To eliminate animal disturbances (e.g., grazing and trampling), we conducted litter decomposition experiments on 30 long‐term large herbivore exclusion plots from July 2022 to October 2023. The average annual precipitation and temperature from 2022 to 2023 in this study area are shown in Appendix S1. Each plot is 10 m × 10 m in size with a 1.6 m height of the surrounding fence. They are at least 1 km apart and have similar environmental conditions, where large animals have been excluded since 2019. The rate of decomposition of leaf litter was measured using the litterbag method. We placed 12 litterbags within each enclosure on July 15–17, 2022: 4 bags of leaf litter (10 g of leaves per litterbag), four bags of sika deer feces and litter mixture (5 g of deer feces + 5 g of leaves), and four bags of cattle feces and litter mixture (5 g of cattle feces +5 g of leaves) (Appendix S2). For leaf litter collection, we laid a trap that was 2.25 m^2^ in size and shook broad‐leaved trees to gather recently senesced leaves near the study sites in July 2022. This harvest was a representative mixture from the five dominant native tree species in the local community, as Liao et al. (2022) reported. The dominant species were *Quercus mongolica*, *Syringa reticulata* subsp., *amurensis*, *Acer pictum* Thunb, *Fraxinus chinensis* subsp. and *Albizia kalkora*. We removed damaged or diseased tissue, as well as nonleaf material. We collected 30 kg of freshly mixed leaves then they were dried for 72 h at 65°C in a laboratory oven. After they were completely mixed by hand, the dried leaf litter was cut into approximately 3 cm long pieces to ensure that the degree of fragmentation of the leaf litter and the proportion of each plant species were consistent after thorough mixing. Leaf litter samples weighing 300 g were used to assess the original litter mass and chemical characteristics. The remaining portion was placed into litterbags for the field decomposition experiments. Litterbags (15 cm × 20 cm) were made using a fiberglass screen (1 mm^2^ mesh openings). Fresh cattle feces were randomly collected from a grazed forest. Sika deer feces were randomly collected from areas with a high density of sika deer. The feces were dried for 72 h at 65°C in a laboratory oven. Fecal samples of 300 g (150 g cattle and 150 g deer) were used to determine the contents of C, N and P in the feces. Twelve litterbags were placed above ground in each enclosure (Appendix S3), one for each collection date, for a total of 4 harvest dates × 3 litter types × 30 replicate plots = 360 litterbags in total. Litterbags were fixed to the ground surface via metal pins to prevent them from moving and were retrieved at 120, 240, 325 and 415 days. When installation them, the litter on the surface layer of the enclosure was carefully removed, and the litterbags were installation parallel to the enclosure without overlapping each other. The fallen leaves were laid flat inside the net bag as much as possible, make it fully contact with the humus layer and approach the natural decomposition state as closely as possible.

### 2.3 Laboratory Analysis

Once the litterbags were harvested, the remaining litter was removed from the bag and carefully cleaned of soil and other extraneous materials. The remaining litter in each bag was oven dried at 65°C for 72 h, weighed, and then milled to measure its C, N, and P concentrations. The total C and N concentrations of the leaf materials were measured via a Vario EL III elemental analyzer (Yang et al. 2022). To determine the total P concentrations, litter samples were initially digested with H_2_SO_4_‐H_2_O_2_, while dry soil samples were digested with H_2_SO_4_‐HClO_4_. Total P concentrations were then quantified following the kjeldahl acid‐digestion method and molybdenum‐blue method by using an auto element analyzer (Smartchen 450, AMS, Guidonia, Italy). Lignin and cellulose content were assessed using the Vaieretti et al. (2013) approach. The tannin concentrations in litter were determined using the ultraviolet and visible spectrum methods. Polysaccharide concentrations in litter were measured using 3,5‐dinitrosalicylic acid colorimetry. The total phenols in litter were determined using Folin‐C: iocalteu colorimetry. We also calculated C/N ratio, C/P ratio, and N/P ratios.

### 2.4 Statistical Analyses

Litter mass loss was calculated by subtracting the initial mass from the sampled mass at each sampling time and dividing the difference by the initial mass (Bohara et al. 2020). The remaining litter mass at time *t* was calculated as the difference between 100% initial litter mass and % litter mass loss at time *t* (Bohara et al. 2020).

The coefficient of litter decomposition (*k*, day^−1^) is adopted to represent litter dynamics (Cai et al. 2020; Makkonen et al. 2012; Zhang et al. 2019). The dry litter mass was weighed individually, and mass loss was calculated via the decay model of Olson (1963) according to the following equation:

(1) $$y={\mathrm{ae}}^{-\mathrm{kt}}$$

where *a* is the correction factor, *k* is the decomposition coefficient (day^−1^), and *y* is the remaining mass (%) at time *t* (days).

Nutrient release by the litter was calculated via the following formula (Guo and Sims 1999):

(2) $$R_{t}=\frac{X_{0}\times C_{0}-X_{t}\times C_{t}}{X_{0}\times C_{0}}\times 100$$

where *C*_0_ is the initial litter nutritional content (g g^−1^), *C*_*t*_ is the nutrient concentration (g g^−1^) in the remaining litter, *X*_0_ is the initial mass at litterbags, *X*_*t*_ is the remaining weight at time *t*, and *R*_*t*_ is the nutrient release (%).

A simple *t*‐test was used to determine the differences in initial fecal chemical properties between sika deer and cattle. Analysis of variance (ANOVA) was used to examine the differences in mass loss and nutrient release of the leaf litter among the groups supplemented with sika deer and cattle feces and the control group at the same decomposition time. Two‐way ANOVA was used to compare the effects of the two large herbivores on the remaining mass and decomposition constants *k* and their interactions. The Kruskal–Wallis test was used to examine the effects of the two large herbivores on nutrient elements in the litter. All effects were regarded as significant at *p* < 0.05.

## 3 Results

### 3.1 Initial Fecal and Litter Chemical Characteristics

The initial sika deer fecal concentrations of total C, total N, and total P were 520 ± 150.85, 31.64 ± 5.44 and 1.24 ± 0.33 g kg^−1^, respectively. The initial cattle fecal concentrations of total C, total N, and total P were 336.47 ± 86.11, 16.73 ± 1.51, and 4.75 ± 1.02 g kg^−1^, respectively (Table 1). The sika deer feces presented significantly higher C (*p* < 0.001) and N (*p* < 0.001) concentrations than the cattle feces (Appendix S4). In contrast, total P was significantly lower in sika deer feces than in cattle feces (*p* < 0.001).

**TABLE 1:** Results of initial fecal chemical properties (*n* = 30) in the Northeast Tiger and Leopard National Park.

Variables	Fecal type	Means	Max	Min	Standard deviations
C (g kg^−1^)	Deer	520.41	882.75	255.00	150.85
	Cattle	336.47	630.53	238.07	86.11
N (g kg^−1^)	Deer	31.64	38.64	16.91	5.44
	Cattle	16.73	19.87	12.99	1.51
P (g kg^−1^)	Deer	1.24	1.73	1.02	0.33
	Cattle	4.75	4.18	5.81	1.02

The initial litter concentrations of total C, total N, total P, lignin, cellulose, tannin, polysaccharide, total phenols, the C/N ratio, the C/P ratio, and the N/P ratio were 480 ± 102.39, 19.82 ± 1.32, 1.45 ± 0.08, 247.37 ± 30.84, 34.15 ± 11.15, 7.08 ± 1.99, 22.68 ± 6.50, 20.08 ± 5.47 g kg^−1^, 24.64 ± 6.58, 325.31 ± 58.53 and 13.62 ± 0.25, respectively (Table 2).

**TABLE 2:** Results of initial litter chemical properties (*n* = 30) in the Northeast Tiger and Leopard National Park.

Variables	Means	Max	Min	Standard deviations
C (g kg^−1^)	480.98	882.00	321.2	102.39
N (g kg^−1^)	19.82	24.66	16.6	1.32
P (g kg^−1^)	1.45	1.62	1.19	0.08
Lignin (g kg^−1^)	247.37	399.29	191.24	30.84
Cellulose (g kg^−1^)	34.15	66.67	16.3	11.15
Tannin (g kg^−1^)	7.08	12.39	2.43	1.99
Polysaccharide (g kg^−1^)	22.68	42.42	11.57	6.50
Total phenols (g kg^−1^)	20.08	52.9	10.26	5.47
C/N	24.64	77.67	19.06	6.58
C/P	325.31	545.01	70.38	58.53
N/P	13.62	15.24	13.08	0.25

### 3.2 Effects of Fecal Addition on the Decomposition Rate

**FIGURE 2:**
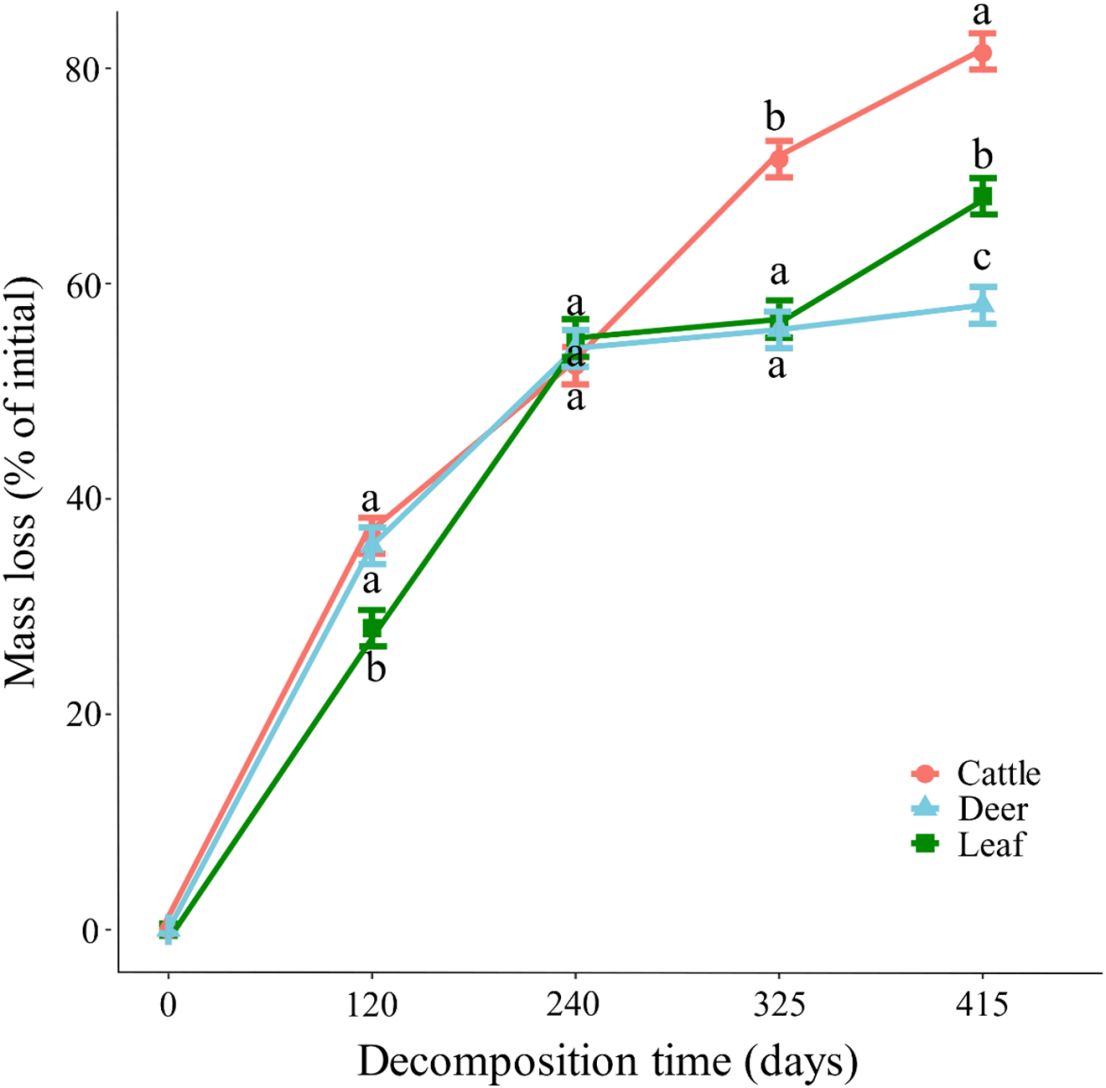
Trajectory of the effects of the addition of cattle feces (red circles), the addition of sika deer feces (blue triangles) and the control treatment (green quadrates) on the loss of litter mass (%) over 415 days of field incubation in a Northeast Asian temperate forest (NTLNP). Different letters in the plot indicate significant differences (*p* < 0.05). The error bars represent the standard deviations of the means (*n* = 30).

**FIGURE 3:**
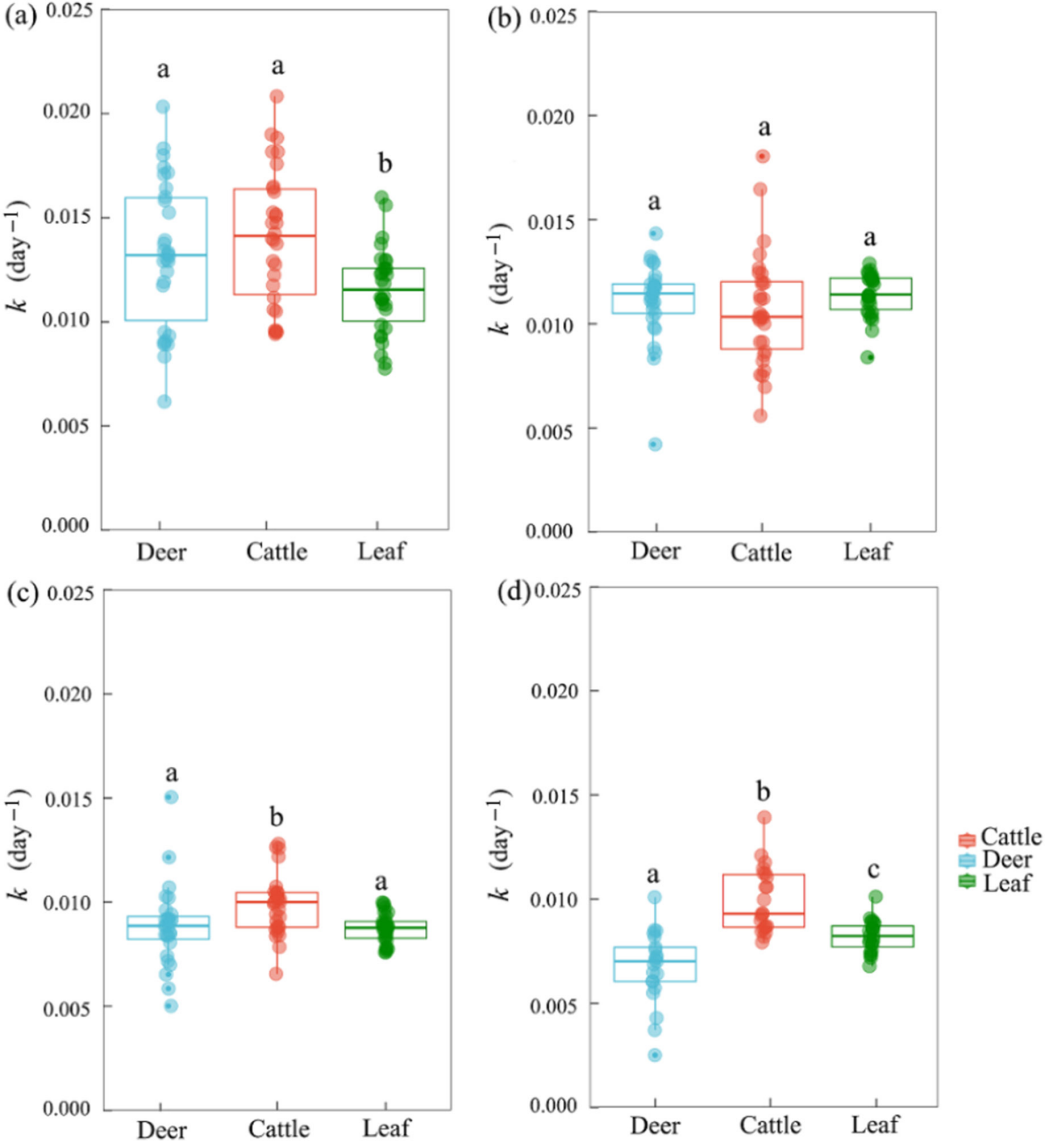
Decomposition constants (*k*, day^−1^) at different decomposition periods with the addition of feces alone and leaf litter alone. (a) Decomposing for 120 days, (b) decomposing for 240 days, (c) decomposing for 325 days, and (d) decomposing for 415 days. Different letters in the plot indicate significant differences (*p* < 0.05).

ANOVA revealed significant effects of fecal addition type, sampling time, and their interactions on the litter mass loss and decomposition constants (*k*, day^−1^) across the study period (all *p* < 0.001, Table 3). At the end of decomposition, adding sika deer feces considerably decreased litter mass loss by 23.43% (*p* < 0.05), while adding cattle feces significantly increased it by 10.12% (*p* < 0.05, Figure 2). During the study period, the rate of litter decomposition decreased gradually across all the treatments as the decomposition time increased, although the decomposition rate significantly differed among the treatments. Compared with the results of the control group (no addition of feces), the addition of cattle dung significantly increased *k* (*p* < 0.05), whereas the addition of sika deer feces decreased *k* (*p* < 0.05, Figure 3).

**TABLE 3:** *F* and *p* values for repeated‐measures ANOVA of the main effects of fecal addition type, sampling time, and their interaction on the litter mass loss and decomposition constants (*k*, day^−1^).

	df	*F*	*p*		df	*F*	*p*
Litter mass loss (% of initial)				Decomposition constants (*k*)			
Type	2	27.89	< 0.001	Type	2	9.345	< 0.001
Time	3	199.48	< 0.001	Time	3	76.73	< 0.001
Type × Time	6	11.73	< 0.001	Type × Time	6	5.946	< 0.001

### 3.3 Effects of Fecal Addition on C, N, and P Release

**FIGURE 4:**
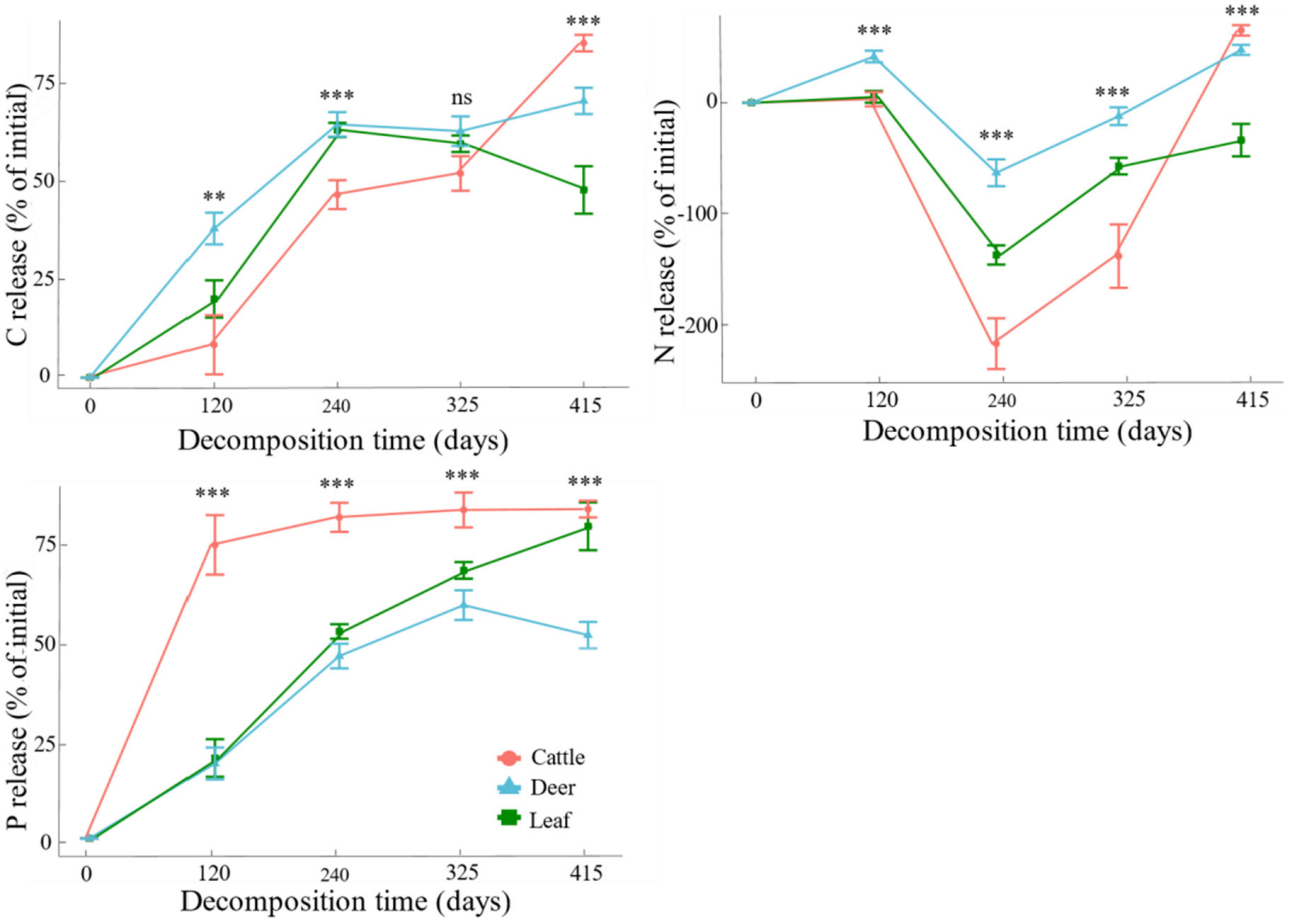
Dynamics of C, N, and P under the different treatments during the entire experimental period. Asterisks indicate that the difference between the specified fecal treatments and the control was significant (*p* < 0.05), with ** < 0.01 and *** < 0.001. The error bars represent the standard deviations of the means (*n* = 30).

**FIGURE 5:**
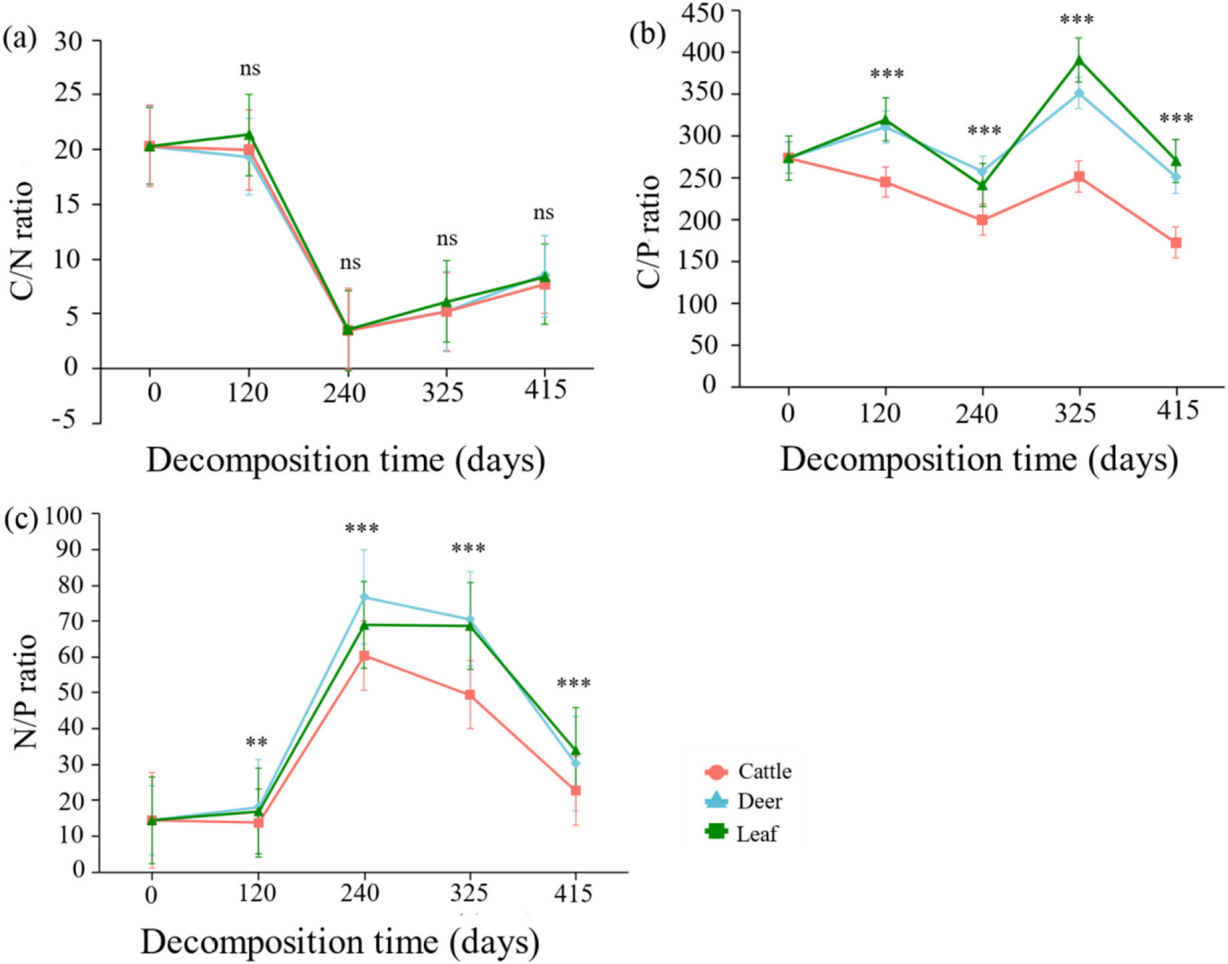
Dynamics of the C/N ratio, C/P ratio, and N/P ratio under the different treatments during the entire experimental period. Asterisks indicate that the difference between the specified fecal treatments and the control was significant (*p* < 0.05), with ** < 0.01 and *** < 0.001. The error bars represent the standard deviations of the means (*n* = 30).

There was a significant difference in C, N and P release under the same decomposition time but with different treatment types (*p* < 0.05, Figure 4). Between 0 and 120 days, the average C release from cattle and sika deer feces increased by 37.45% and 22.69%, respectively, compared with the results of the control group. In the middle of decomposition, N accumulated in all three treatment groups, and the presence of cattle feces significantly promoted N accumulation compared with that in deer (the average N accumulation increased by 154%). Throughout the decomposition period, in contrast to the control group, cattle feces accelerated the loss of P (4.35%), but deer feces reduced the release of P (27.55%). At the end of decomposition (415 days), the litter C/N ratio was no significant difference (Figure 5a). The C/P decreased by 7.20% and 36.19% in regard to the sika deer and cattle feces, respectively, during the study period compared with that in the control group (270.48 ± 57.44, Kruskal–Wallis test, *p* < 0.05, Figure 5b). The N/P ratio decreased by 10.72% and 32.97% in regard to the sika deer and cattle feces, respectively, during the study period compared with that in the control group (34.00 ± 4.99, Kruskal–Wallis test, *p* < 0.05, Figure 5c).

## 4 Dicussion

Large herbivores may affect nutrient cycling by processing plants into urine and feces and influencing litter decomposition (Mancilla‐Leytón, Sánchez‐Lineros, and Vicente 2013). The type of grazing animal, the quality and composition of the forage consumed, and excreta could affect the nutrient cycling speed and efficiency (de Sousa et al. 2024). We provide experimental evidence that wild ungulates and cattle have different effects on litter decomposition and nutrient cycling via the excrement decomposition process. The addition of sika deer feces greatly slowed litter decomposition, but the addition of cattle feces significantly accelerated litter decomposition. At the end of decomposition, the addition of cattle feces resulted in a much greater release rate of C than did the addition of sika deer feces. The addition of feces increased the release of N. The addition of feces enhanced the release of P, whereas the addition of feces from sika deer reduced the release of P.

### 4.1 Response of Litter Decomposition to Fecal Addition

Litter decomposition is a process that can be divided into two stages. In the initial phase, soluble litter molecules (simple sugars, carbohydrates, fatty acids, amino acids, etc.) are rapidly consumed by soil microorganisms or are leached or volatilized into the environment (Berg 2014). This stage usually has the greatest decomposition rates, resulting in rapid plant material loss in a short time (Naeem et al. 2022). The second phase commonly has low decomposition rates because of the synthesis of lignin‐like molecules and greater concentration of recalcitrant materials (fibers, lignin, etc.), which are not decomposed in the initial phase. These compounds need specialized microbial intervention for their decomposition (cellulolytic bacteria or fungi) and require a longer process, ending with the formation of long‐lasting stable compounds in the soil (Garzon et al. 2024; Guo and Sims 1999). Our results showed that the decomposition of the litter in the Northeast Asian temperate forest was faster before 240 days and slower after 240 days, which is in line with the general trend. Berg (2014) also reported that the litter decomposition rate varied at different stages. In the early phases of decomposition (the autumn and winter seasons) in our study, water may have formed ice crystals in the litter, expanding the pores and cracks of the litter. They had a crushing effect on the litter and increased the surface area of the reaction, making it easier for microorganisms and enzymes to enter the interior of the litter for decomposition (Yang and Wang 2019).

Our results revealed that the initial N content in the feces of sika deer was approximately twice that in domestic cattle feces, while P content was roughly four times lower. This is due to the difference in dietary composition between sika deer and cattle. Wu et al. (2023) found that the diets of cattle and sika deer overlapped greatly, but the proportions of each food differed in our study area. Rosaceae and Betulaceae are the primary dietary sources of sika deer, with a higher proportion consumed than cattle, and the crude protein of primary food is mainly made up of C and N. *Pilea pumila* (L.) A. Gray of the Urticaceae was consumed in a much higher proportion by cattle than sika deer. *Pilea pumila* (L.) A. Gray has a high P concentration compared to other plants, resulting in much more P in cattle feces than in sika deer feces.

We found that the decomposition rate of litter varied considerably between different fecal types (Figure 3). Litter decomposition in forest ecosystems in a natural state is influenced by complex factors. Sika deer feces with higher N contents had a strong inhibitory effect on litter decomposition, possibly because the input of N exceeded a critical value (Aber and Melillo 1982). Aber and Melillo (1982) reported an inverse linear relationship between the remaining mass and N concentration in litter, which has been supported by many studies of litter decomposition (Mancilla‐Leytón, Sánchez‐Lineros, and Vicente 2013). Furthermore, after N addition was enriched in fallen leaves, it may have exceeded this critical threshold, disrupted the initial N balance and reduced the decomposition efficiency of the decomposer, thereby slowing litter decomposition (Manning et al. 2008; Zhang et al. 2022). The presence of sika deer feces in N‐rich leaves results in the formation of complex chemical macromolecules with phenolics and impedes the degradation of lignin and cellulose, which delays the decomposition of litter (Tu et al. 2011). Another possibility is that the N complex used for fertilization increases the acidity of the pH, which may also decrease the decomposition rate. Moreover, soil organic C may be more difficult for microorganisms to use in high‐N soils than in low‐N soils because increased N availability may suppress basidiomycetes and the activity of ligninolytic and cellulitis enzymes involved in recalcitrant C decomposition, such as phenol oxidase and peroxidase (Carreiro et al. 2000; Sirulnik et al. 2007). Carreiro et al. (2000) reported that adding N delayed the decomposition of litter by inhibiting the lignin‐degrading enzymes production, which supports the findings of this study. In line with previous research, wild yak (*B. grunniens*) grazing increased mass loss and shortened the decomposition time of both feces and litter on the Qinghai‐Tibet Plateau (Sun et al. 2018). Jiang et al. (2024) reported that deer grazing has a suppressive influence on the decomposition of litter, whereas livestock grazing can accelerate it. Deer selective foraging is expected to lower the quality and quantity of woody plants (Chollet et al. 2021), as well as the availability of carbohydrates accessible to roots and ectomycorrhizal associations (Rossow, Bryant, and Kielland 1997; Stark, Männistö, and Smolander 2010), impeding the decomposition rate of litter. We also discovered that domestic cattle grazing promoted litter decomposition, whereas sika deer impeded it.

### 4.2 Litter Nutrient Dynamics Under Different Fecal Addition Treatments

The study of domestic animals and/or forest ecosystems has contributed significantly to our understanding of the impact of dung and urine deposition by large herbivores on nitrogen cycling (Frank and Groffman 1998; Luo et al. 2010; Walker, Anderson, and Fugal 2015). We demonstrated that, in the temperate forests we studied, cattle and sika deer feces decomposed faster and released a greater proportion of C and N than plant litter (Figure 4). This may be explained by the presence of labile nutrients in cattle and sika deer feces, which increase the development of microbial communities and increase rates of nutrient cycling (Chollet et al. 2021). Nutrient release from plant residues into soil is complicated and vulnerable to biological degradation (Sun et al. 2018). Decomposers transfer organic C and N into inorganic matter (e.g., CO_2_ and NO^3−^) through respiration, immobilization or mineralization. Plants absorb and utilize inorganic matter (Chen et al. 2011), while also providing energy to decomposers (Manzoni et al. 2008). Like the results of mass loss in the feces and litter, the C in the decomposing litter was released throughout the entire decomposition cycle in all three treatment groups. During the early and middle stages of decomposition, the litter contained more easily degradable substances that could be directly utilized by decomposers. Therefore, a large loss of water‐soluble C resulted in rapid leaching of litter C during the first and intermediate phases of decomposition (Yang et al. 2019). After 325 days of decomposition, the presence of sika deer and cattle excrement dramatically accelerated the release rate of C.

N release is a more complex process than is litter decomposition. N release can involve any one or both processes of N immobilization and mineralization, with the former resulting in the accumulation of N in the litter and the latter causing the release of N from the litter (Sun et al. 2018). Mineral elements, notably N, play an important role in controlling decomposition rates in organic materials. Ågren, Bosatta, and Magill (2001) reported that the magnitude and even the sign of these effects vary and that the underlying mechanisms remain unclear. Leaching and microbial degradation are the main processes influencing nutrient changes during litter decomposition. Leaching is a key process that causes nutrient loss. Microbial degradation processes fix nitrogen, which mostly exists in the form of proteins, leading to nitrogen accumulation. Previous research has indicated that the supply of nutrient N influences litter decomposition (Berg 2014), and that nitrogen fertilizer may affect the decomposing bacterial community or produce abiotic effects with intermediates in process of microbial degradation and synthesis, leading to a decrease in litter decomposition rate (Hobbie 2008). Our study revealed N enrichment in the middle decomposition stage of litter, which could be attributed to the fact that the litter used in this study included fresh leaves and that the N content in fresh litter was relatively low. In addition, the results align with those of earlier research (Aber and Melillo 1980; Hefting et al. 2005), and N accumulation in the litter may have occurred because the initial C/N in the three groups was less than 25, which could not meet the needs of microbial decomposition metabolism in the litter, forcing microorganisms to absorb a certain amount of N from the soil, leading to N enrichment in the litter. Furthermore, the study area was in a Northeast Asian temperate forest with considerable snowfall in the winter, and a large amount of N in snow cover is released from N sources such as soluble inorganic nitrogen, dust particles, and organic pollutants. A lower soil temperature reduces microbial activity and increases the litter N content. Under conditions of litter nutrient limitation, up to 50% of nutrients (e.g., total N) in decomposing litter can be imported from the underlying soil via fungal hyphae (Shilenkova and Tiunov 2013). Our results revealed that N enrichment due to the presence of cattle feces was substantially greater than that due to the presence of sika deer feces. The initial N concentration was approximately twofold greater in the presence of sika deer feces than in the presence of cattle feces (Appendix S4), which most likely led to more N being transferred from the topsoil to the decomposing leaf litter (Shilenkova and Tiunov 2013). Results from Shilenkova and Tiunov (2013) are consistent with our results; they discovered that a significant amount of the nitrogen fixed in the soil was in the form of nonsoluble components in microbial tissues such as fungal hyphae.

Most studies have indicated that P is released during the litter decomposition process (Feng, Xue, and Chen 2018; Moore et al. 2006; Zhang et al. 2022). P release is greatest during the litter decomposition early stage, which may be due to rapid loss of litter mass. During the process of litter decomposition, P exists in a soluble form and is easily leached (Moore et al. 2006). This study revealed that P release was achieved in the three treatment groups, with the addition of sika deer and cattle feces having differing impacts on P release. Sika deer reduced P release, whereas cattle accelerated P release. Many studies have shown that P release and accumulation are affected by thresholds.

When both the C/N and C/P ratios are less than 40 and 480, respectively (Chen, Gong, and Liu 2018; Parton et al. 2007; Ren et al. 2024), the litter P is more easily released. In this study, the C/N ratio and C/P ratio were both within the ranges mentioned above. The P of the litter rapidly increased between 0 and 240 days of decomposition, and the increase was particularly noticeable during this period. During autumn and winter, the temperature was relatively low, and the microbial biomass and activity were low; thus, large amounts of P were not required for microbial survival, resulting in increased litter P levels. In the litter decomposition later stages, P release caused by the addition of cattle feces tended to be slow because the proportion of difficult‐to‐decompose compounds such as cellulose and lignin increased, resulting in a slow increase in the cumulative release rate of elements (Moore et al. 2006).

## 5 Conclusions and Management Implications

Overall, our findings suggest that the feces of the two large herbivores had nonequivalent effects on litter decomposition. Our field experiment reveals that the addition of sika deer feces greatly reduced litter decomposition, whereas livestock grazing increased it. The presence of cattle and deer excrement significantly accelerated the release of C. The elemental changes during decomposition revealed that N was the least released nutrient, suggesting that litter serves as an N stock, whereas the rapid release of the other nutrients (C and P) indicates that the litter is a potential source of C and P. These results have implications for C, N, and P turnover in temperate forest ecosystems under the future coexistence of wild herbivores and livestock, as well as laying the groundwork for understanding the impact of the litter decomposition process on the material cycle and energy flow of forest ecosystems to some extent.

Livestock grazing in protected forests has become a controversial management practice, both globally and within China. The presence/abundance of cattle contributes to habitat degradation and loss of mammal diversity in the Northeast Tiger and Leopard National Park (Feng et al. 2021; Li et al. 2022; Wang et al. 2016). Thus, to reduce human‐wildlife conflict, the removal of grazing rights from core conservation zones for tigers and leopards is possible. However, integrating livestock and wildlife can provide complementary ecological (e.g., by increasing forage quality for some wildlife species) and economic benefits (e.g., by increasing local food and tourism incomes) (Keesing et al. 2018), and fences can be viewed as potential conservation tools if managed properly for wildlife survival (Vélez et al. 2024). For example, fences are effective at selectively excluding cattle and increasing the encounter rates of wild ungulates in forest (Vélez et al. 2024). Given that smaller body sizes of wildlife compared to domestic animals such as cattle, we suggest that implementation of wildlife‐permeable fences in some forest patches in this park could be considered in the future (Smith, King, and Allen 2020; Vélez et al. 2024). Considering the goal of restoring trophic interactions and ecosystem functions in this park, low‐intensity grazing in noncore management zones may be necessary to improve forest nitrogen cycling and energy flow. However, if there is evidence of serious degradation caused by years of overgrazing, grazing reduction or exclusion may be a more appropriate option in the overall national park.

The effects of large ungulates on litter decomposition vary between monospecific and mixed litters; thus, future studies need to consider the effects of wild ungulates and livestock activities (e.g., grazing and trampling) on the decomposition rate of monospecific or mixed litters. Additionally, microbial decomposition is an important pathway in the process of litter decomposition; hence, research on litter decomposition is inextricably linked to microorganism experiments. We do not fully understand how this relates to the observed changes in decomposition rates because decomposer communities have not been studied; however, this issue surely warrants further study. We recognize the effects we have measured represent short‐term responses to wild ungulates and cattle excrement in the forest. Future studies with longer‐term decomposition experiments are needed to monitor nutrient turnover in Tiger and Leopard National Park in the context of the coexistence of wild herbivores and livestock.

## Author Contributions

**Yongchun Hu:** data curation (equal), investigation (equal), methodology (equal), visualization (equal), writing – original draft (equal), writing – review and editing (equal). **Jiawei Feng:** data curation (equal), writing – original draft (equal), writing – review and editing (equal). **Hongfang Wang:** visualization (equal), writing – review and editing (equal). **Jianping Ge:** conceptualization (equal), writing – original draft (equal), writing – review and editing (equal). **Tianming Wang:** conceptualization (equal), funding acquisition (equal), writing – original draft (equal), writing – review and editing (equal).

## Conflicts of Interest

The authors declare no conflicts of interest.

## Supporting information


Appendix S1‐S4.

## Data Availability

All data was made publicly available in a public repository at https://doi.org/10.57760/sciencedb.11463.

## References

[ece370529-bib-0001] Aber, J. D. , and J. M. Melillo . 1980. “Litter Decomposition: Measuring Relative Contributions of Organic Matter and Nitrogen to Forest Soils.” Canadian Journal of Botany‐Revue Canadienne De Botanique 58: 416–421. 10.1139/b80-046.

[ece370529-bib-0002] Aber, J. D. , and J. M. Melillo . 1982. “Nitrogen Immobilization in Decaying Hardwood Leaf Litter as a Function of Initial Nitrogen and Lignin Content.” Canadian Journal of Botany‐Revue Canadienne De Botanique 60: 2263–2269. 10.1139/b82-277.

[ece370529-bib-0003] Ågren, G. I. , E. Bosatta , and A. H. Magill . 2001. “Combining Theory and Experiment to Understand Effects of Inorganic Nitrogen on Litter Decomposition.” Oecologia 128: 94–98. 10.1007/s004420100646. 28547095

[ece370529-bib-0004] Banegas, N. , A. S. Albanesi , R. O. Pedraza , and D. A. Dos Santos . 2015. “Non‐Linear Dynamics of Litter Decomposition Under Different Grazing Management Regimes.” Plant and Soil 393: 47–56. 10.1007/s11104-015-2472-y.

[ece370529-bib-0005] Bardgett, R. D. , and D. A. Wardle . 2003. “Herbivore‐Mediated Linkages Between Aboveground and Belowground Communities.” Ecology 84: 2258–2268. 10.1890/02-0274.

[ece370529-bib-0006] Beer, C. , M. Reichstein , E. Tomelleri , et al. 2010. “Terrestrial Gross Carbon Dioxide Gptake: Global Distribution and Covariation With Climate.” Science 329: 834–838. 10.1126/science.1184984. 20603496

[ece370529-bib-0007] Berg, B. 2014. “Decomposition Patterns for Foliar Litter—A Theory for Influencing Factors.” Soil Biology & Biochemistry 78: 222–232. 10.1016/j.soilbio.2014.08.005.

[ece370529-bib-0008] Bernes, C. , B. Macura , B. G. Jonsson , et al. 2018. “Manipulating Ungulate Herbivory in Temperate and Boreal Forests: Effects on Vegetation and Invertebrates. A Systematic Review.” Environmental Evidence 7: 13. 10.1186/s13750-018-0125-3.

[ece370529-bib-0009] Bobbink, R. , M. Hornung , and J. G. M. Roelofs . 1998. “The Effects of Air‐Borne Nitrogen Pollutants on Species Diversity in Natural and Semi‐Natural European Vegetation.” Journal of Ecology 86: 717–738. 10.1046/j.1365-2745.1998.8650717.x.

[ece370529-bib-0010] Bohara, M. , K. Acharya , S. Perveen , et al. 2020. “ *In Situ* Litter Decomposition and Nutrient Release From Forest Trees Along an Elevation Gradient in Central Himalaya.” Catena 194: e104698. 10.1016/j.catena.2020.104698.

[ece370529-bib-0011] Cai, A. D. , N. J. Chang , W. J. Zhang , et al. 2020. “The Spatial Patterns of Litter Turnover Time in Chinese Terrestrial Ecosystems.” European Journal of Soil Science 71: 856–867. 10.1111/ejss.12922.

[ece370529-bib-0012] Cai, A. D. , G. P. Liang , W. Yang , et al. 2021. “Patterns and Driving Factors of Litter Decomposition Across Chinese Terrestrial Ecosystems.” Journal of Cleaner Production 278: 123964. 10.1016/j.jclepro.2020.123964.

[ece370529-bib-0013] Carreiro, M. M. , R. L. Sinsabaugh , D. A. Repert , and D. F. Parkhurst . 2000. “Microbial Enzyme Shifts Explain Litter Decay Responses to Simulated Nitrogen Deposition.” Ecology 81: 2359–2365. 10.1890/0012-9658(2000)081[2359:Meseld]2.0.Co;2.

[ece370529-bib-0014] Cebrian, J. 1999. “Patterns in the Fate of Production in Plant Communities.” American Naturalist 154: 449–468. 10.1086/303244. 10523491

[ece370529-bib-0015] Chen, W. W. , B. Wolf , N. Brüggemann , K. Butterbach‐Bahl , and X. H. Zheng . 2011. “Annual Emissions of Greenhouse Gases From Sheepfolds in Inner Mongolia.” Plant and Soil 340: 291–301. 10.1007/s11104-010-0367-5.

[ece370529-bib-0016] Chen, X. , L. Gong , and Y. T. Liu . 2018. “The Ecological Stoichiometry and Interrelationship Between Litter and Soil Under Seasonal Snowfall in Tianshan Mountain.” Ecosphere 9: e02520. 10.1002/ecs2.2520.

[ece370529-bib-0017] Chollet, S. , M. Maillard , J. Schörghuber , S. J. Grayston , and J. L. Martin . 2021. “Deer Slow Down Litter Decomposition by Reducing Litter Quality in a Temperate Forest.” Ecology 102: e03235. 10.1002/ecy.3235. 33098575

[ece370529-bib-0018] Cornwell, W. K. , J. H. C. Cornelissen , K. Amatangelo , et al. 2008. “Plant Species Traits Are the Predominant Control on Litter Decomposition Rates Within Biomes Worldwide.” Ecology Letters 11: 1065–1071. 10.1111/j.1461-0248.2008.01219.x. 18627410

[ece370529-bib-0019] de Sousa, M. , L. C. Muniz , V. X. D. Apolinário , et al. 2024. “Nitrogen Fertilization Increased Grass Litter Decomposition in a Tropical Agroforestry System.” Agroforestry Systems 98: 995–1008. 10.1007/s10457-024-00968-x.

[ece370529-bib-0020] Dou, H. L. , H. T. Yang , J. L. D. Smith , L. M. Feng , T. M. Wang , and J. P. Ge . 2019. “Prey Selection of Amur Tigers in Relation to the Spatiotemporal Overlap With Prey Across the Sino‐Russian Border.” Wildlife Biology 2019: 1–11. 10.2981/wlb.00508.

[ece370529-bib-0021] Du, N. N. , W. R. Li , L. P. Qiu , Y. J. Zhang , X. R. Wei , and X. C. Zhang . 2020. “Mass Loss and Nutrient Release During the Decomposition of Sixteen Types of Plant Litter With Contrasting Quality Under Three Precipitation Regimes.” Ecology and Evolution 10: 3367–3382. 10.1002/ece3.6129. 32273994 PMC7141022

[ece370529-bib-0022] Feng, H. F. , L. Xue , and H. Y. Chen . 2018. “Responses of Decomposition of Green Leaves and Leaf Litter to Stand Density, N and P Additions in Acacia Auriculaeformis Stands.” European Journal of Forest Research 137: 819–830. 10.1007/s10342-018-1142-z.

[ece370529-bib-0023] Feng, J. W. , Y. F. Sun , H. L. Li , et al. 2021. “Assessing Mammal Species Richness and Occupancy in a Northeast Asian Temperate Forest Shared by Cattle.” Diversity and Distributions 27: 857–872. 10.1111/ddi.13237.

[ece370529-bib-0024] Frank, D. A. , and P. M. Groffman . 1998. “Denitrification in a Semi‐Arid Grazing Ecosystem.” Oecologia 117: 564–569. 10.1007/s004420050693. 28307682

[ece370529-bib-0025] Fu, Y. W. , M. Y. Tan , Y. A. Gong , et al. 2022. “Wild Boar Survives in a Landscape That Prohibits Anthropogenic Persecution.” Frontiers in Ecology and Evolution 10: 820915. 10.3389/fevo.2022.820915.

[ece370529-bib-0026] Fukasawa, Y. , S. Katsumata , A. S. Mori , T. Osono , and H. Takeda . 2014. “Accumulation and Decay Dynamics of Coarse Woody Debris in a Japanese Old‐Growth Subalpine Coniferous Forest.” Ecological Research 29: 257–269. 10.1007/s11284-013-1120-3.

[ece370529-bib-0027] Garzon, J. , J. M. B. Vendramini , M. L. Silveira , et al. 2024. “Aeschynomene Overseeding and Nitrogen Fertilization Effects in Bahiagrass Litter Decomposition.” Agronomy Journal 116: 1455–1465. 10.1002/agj2.21561.

[ece370529-bib-0028] Giorgis, M. A. , A. M. Cingolani , I. Teich , and M. Poca . 2020. “Can Livestock Coexist With Forests in Mountains of Central Argentina? Setting Thresholds for a Land Sharing Landscape.” Forest Ecology and Management 457: 117728. 10.1016/j.foreco.2019.117728.

[ece370529-bib-0029] Guo, L. B. , and R. E. H. Sims . 1999. “Litter Decomposition and Nutrient Release via Litter Decomposition in New Zealand Eucalypt Short Rotation Forests.” Agriculture Ecosystems & Environment 75: 133–140. 10.1016/s0167-8809(99)00069-9.

[ece370529-bib-0030] Hefting, M. M. , J. C. Clement , P. Bienkowski , et al. 2005. “The Role of Vegetation and Litter in the Nitrogen Dynamics of Riparian Buffer Zones in Europe.” Ecological Engineering 24: 465–482. 10.1016/j.ecoleng.2005.01.003.

[ece370529-bib-0031] Hobbie, S. E. 2008. “Nitrogen Effects on Decomposition: A Five‐Year Experiment in Eight Temperate Sites.” Ecology 89: 2633–2644. 10.1890/07-1119.1. 18831184

[ece370529-bib-0032] Jiang, A. , T. D. Mipam , L. H. Jing , et al. 2024. “Large Herbivore Grazing Accelerates Litter Decomposition in Terrestrial Ecosystems.” Science of the Total Environment 922: 171288. 10.1016/j.scitotenv.2024.171288. 38423309

[ece370529-bib-0033] Kasahara, M. , S. Fujii , T. Tanikawa , and A. S. Mori . 2016. “Ungulates Decelerate Litter Decomposition by Altering Litter Quality Above and Below Ground.” European Journal of Forest Research 135: 849–856. 10.1007/s10342-016-0978-3.

[ece370529-bib-0034] Kastovská, E. , J. Mastny , and M. Konvicka . 2024. “Rewilding by Large Ungulates Contributes to Organic Carbon Storage in Soils.” Journal of Environmental Management 355: 120430. 10.1016/j.jenvman.2024.120430. 38428182

[ece370529-bib-0035] Keesing, F. , R. S. Ostfeld , S. Okanga , et al. 2018. “Consequences of Integrating Livestock and Wildlife in an African Savanna.” Nature Sustainability 1: 566–573. 10.1038/s41893-018-0149-2.

[ece370529-bib-0036] Li, S. , Z. Y. Hou , J. P. Ge , and T. M. Wang . 2022. “Assessing the Effects of Large Herbivores on the Three‐Dimensional Structure of Temperate Forests Using Terrestrial Laser Scanning.” Forest Ecology and Management 507: 119985. 10.1016/j.foreco.2021.119985.

[ece370529-bib-0037] Liao, C. , C. Y. Long , Q. Zhang , and X. L. Cheng . 2022. “Stronger Effect of Litter Quality Than Micro‐Organisms on Leaf and Root Litter C and N Loss at Different Decomposition Stages Following a Subtropical Land Use Change.” Functional Ecology 36: 896–907. 10.1111/1365-2435.13999.

[ece370529-bib-0038] Luo, C. Y. , G. P. Xu , Z. G. Chao , et al. 2010. “Effect of Warming and Grazing on Litter Mass Loss and Temperature Sensitivity of Litter and Dung Mass Loss on the Tibetan Plateau.” Global Change Biology 16: 1606–1617. 10.1111/j.1365-2486.2009.02026.x.

[ece370529-bib-0039] Makkonen, M. , M. P. Berg , I. T. Handa , et al. 2012. “Highly Consistent Effects of Plant Litter Identity and Functional Traits on Decomposition Across a Latitudinal Gradient.” Ecology Letters 15: 1033–1041. 10.1111/j.1461-0248.2012.01826.x. 22732002

[ece370529-bib-0040] Mancilla‐Leytón, J. M. , V. Sánchez‐Lineros , and A. M. Vicente . 2013. “Influence of Grazing on the Decomposition of *Pinus Pinea* L. Needles in a Silvopastoral System in Doana, Spain.” Plant and Soil 373: 173–181. 10.1007/s11104-013-1788-8.

[ece370529-bib-0041] Manning, P. , M. Saunders , R. D. Bardgett , et al. 2008. “Direct and Indirect Effects of Nitrogen Deposition on Litter Decomposition.” Soil Biology & Biochemistry 40: 688–698. 10.1016/j.soilbio.2007.08.023.

[ece370529-bib-0042] Manzoni, S. , R. B. Jackson , J. A. Trofymow , and A. Porporato . 2008. “The Global Stoichiometry of Litter Nitrogen Mineralization.” Science 321: 684–686. 10.1126/science.1159792. 18669860

[ece370529-bib-0043] Moore, T. R. , J. A. Trofymow , C. E. Prescott , J. Fyles , and B. D. Titus . 2006. “Patterns of Carbon, Nitrogen and Phosphorus Dynamics in Decomposing Foliar Litter in Canadian Forests.” Ecosystems 9: 46–62. 10.1007/s10021-004-0026-x.

[ece370529-bib-0044] Naeem, I. , X. F. Wu , T. Asif , L. Wang , and D. L. Wang . 2022. “Livestock Diversification Implicitly Affects Litter Decomposition Depending on Altered Soil Properties and Plant Litter Quality in a Meadow Steppe.” Plant and Soil 473: 49–62. 10.1007/s11104-021-05006-8.

[ece370529-bib-0045] Öllerer, K. , A. Varga , K. Kirby , et al. 2019. “Beyond the Obvious Impact of Domestic Livestock Grazing on Temperate Forest Vegetation—A Global Review.” Biological Conservation 237: 209–219. 10.1016/j.biocon.2019.07.007.

[ece370529-bib-0046] Olson, J. S. 1963. “Energy Storage and the Balance of Producers and Decomposers in Ecological Systems.” Science 44: 322–331. 10.2307/1932179.

[ece370529-bib-0047] Parton, W. , W. L. Silver , I. C. Burke , et al. 2007. “Global‐Scale Similarities in Nitrogen Release Patterns During Long‐Term Decomposition.” Science 315: 361–364. 10.1126/science.1134853. 17234944

[ece370529-bib-0048] Pastor, J. , B. Dewey , R. J. Naiman , P. F. McInnes , and Y. Cohen . 1993. “Moose Browsing and Soil Fertility in the Boreal Forests of Isle‐Royale‐National‐Park.” Ecology 74: 467–480. 10.2307/1939308.

[ece370529-bib-0049] Prescott, C. E. , and L. Vesterdal . 2021. “Decomposition and Transformations Along the Continuum From Litter to Soil Organic Matter in Forest Soils.” Forest Ecology and Management 498: 119522. 10.1016/j.foreco.2021.119522.

[ece370529-bib-0050] Pringle, R. M. , J. O. Abraham , T. M. Anderson , et al. 2023. “Impacts of Large Herbivores on Terrestrial Ecosystems.” Current Biology 33: R584–R610. 10.1016/j.cub.2023.04.024. 37279691

[ece370529-bib-0051] Ren, Y. , G. L. Gao , G. D. Ding , Y. Zhang , and P. S. Zhao . 2024. “Patterns and Environmental Drivers of C, N, and P Stoichiometry in the Leaf‐Litter‐Soil System Associated With Mongolian Pine Forests.” Ecology and Evolution 14: e11172. 10.1002/ece3.11172. 38516573 PMC10954427

[ece370529-bib-0052] Rossow, L. J. , J. P. Bryant , and K. Kielland . 1997. “Effects of Above‐Ground Browsing by Mammals on Mycorrhizal Infection in an Early Successional Taiga Ecosystem.” Oecologia 110: 94–98. 10.1007/s004420050137. 28307473

[ece370529-bib-0053] Shilenkova, O. L. , and A. V. Tiunov . 2013. “Soil–Litter Nitrogen Transfer and Changes in δ13C and δ15N Values in Decomposing Leaf Litter During Laboratory Incubation.” Pedobiologia 56: 147–152. 10.1016/j.pedobi.2013.03.004.

[ece370529-bib-0054] Sirulnik, A. G. , E. B. Allen , T. Meixner , and M. F. Allen . 2007. “Impacts of Anthropogenic N Additions on Nitrogen Mineralization From Plant Litter in Exotic Annual Grasslands.” Soil Biology & Biochemistry 39: 24–32. 10.1016/j.soilbio.2006.04.048.

[ece370529-bib-0055] Smith, D. , R. King , and B. L. Allen . 2020. “Impacts of Exclusion Fencing on Target and Non‐Target Fauna: A Global Review.” Biological Reviews 95: 1590–1606. 10.1111/brv.12631. 32725786

[ece370529-bib-0056] Stark, S. , M. K. Männistö , and A. Smolander . 2010. “Multiple Effects of Reindeer Grazing on the Soil Processes in Nutrient‐Poor Northern Boreal Forests.” Soil Biology & Biochemistry 42: 2068–2077. 10.1016/j.soilbio.2010.08.001.

[ece370529-bib-0057] Sun, Y. , X. Z. He , F. J. Hou , Z. F. Wang , and S. H. Chang . 2018. “Grazing Increases Litter Decomposition Rate but Decreases Nitrogen Release Rate in an Alpine Meadow.” Biogeosciences 15: 4233–4243. 10.5194/bg-15-4233-2018.

[ece370529-bib-0058] Swain, M. , S. J. Leroux , and R. Buchkowski . 2023. “Strong Above‐Ground Impacts of a Non‐Native Ungulate Do Not Cascade to Impact Below‐Ground Functioning in a Boreal Ecosystem.” Journal of Animal Ecology 92: 2016–2027. 10.1111/1365-2656.13993. 37565516

[ece370529-bib-0059] Tu, L. H. , H. L. Hu , T. X. Hu , et al. 2011. “Decomposition of Different Litter Fractions in a Subtropical Bamboo Ecosystem as Affected by Experimental Nitrogen Deposition.” Pedosphere 21: 685–695. 10.1016/s1002-0160(11)60171-9.

[ece370529-bib-0060] Vaieretti, M. V. , A. M. Cingolani , N. P. Harguindeguy , and M. Cabido . 2013. “Effects of Differential Grazing on Decomposition Rate and Nitrogen Availability in a Productive Mountain Grassland.” Plant and Soil 371: 675–691. 10.1007/s11104-013-1831-9.

[ece370529-bib-0061] Veen, G. F. , E. L. Fry , F. C. ten Hooven , P. Kardol , E. Morriën , and J. R. De Long . 2019. “The Role of Plant Litter in Driving Plant‐Soil Feedbacks.” Frontiers in Environmental Science 7: 168. 10.3389/fenvs.2019.00168.

[ece370529-bib-0062] Vélez, J. , W. McShea , B. Pukazhenthi , et al. 2024. “Cattle Exclusion Increases Encounters of Wild Herbivores in Neotropical Forests.” Journal of Applied Ecology 61: 2444–2454. 10.1111/1365-2664.14751.

[ece370529-bib-0063] Walker, S. C. , V. J. Anderson , and R. A. Fugal . 2015. “Big Game and Cattle Influence on Aspen Community Regeneration Following Prescribed Fire.” Rangeland Ecology & Management 68: 354–358. 10.1016/j.rama.2015.05.005.

[ece370529-bib-0064] Wang, B. , K. Verheyen , L. Baeten , and P. De Smedt . 2021. “Herb Litter Mediates Tree Litter Decomposition and Soil Fauna Composition.” Soil Biology & Biochemistry 152: 108063. 10.1016/j.soilbio.2020.108063.

[ece370529-bib-0065] Wang, L. , J. Feng , T. Amarsaikhan , et al. 2019. “Forest Cattle Grazing Affects Understory Food Resource of Ungulates in the Eastern Part of the Northeast Tiger and Leopard National Park.” Acta Theriologica Sinica 39: 386–396. 10.16829/j.slxb.150303.

[ece370529-bib-0066] Wang, T. M. , L. M. Feng , P. Mou , et al. 2016. “Amur Tigers and Leopards Returning to China: Direct Evidence and a Landscape Conservation Plan.” Landscape Ecology 31: 491–503. 10.1007/s10980-015-0278-1.

[ece370529-bib-0067] Wang, T. M. , J. A. Royle , J. L. D. Smith , et al. 2018. “Living on the Edge: Opportunities for Amur Tiger Recovery in China.” Biological Conservation 217: 269–279. 10.1016/j.biocon.2017.11.008.

[ece370529-bib-0068] Wei, Y. Q. , Y. J. Zhang , G. W. T. Wilson , et al. 2021. “Transformation of Litter Carbon to Stable Soil Organic Matter Is Facilitated by Ungulate Trampling.” Geoderma 385: 114828. 10.1016/j.geoderma.2020.114828.

[ece370529-bib-0069] Wu, F. , D. Zhu , P. Y. Wen , et al. 2023. “Domestic Cattle in a National Park Restricting the Sika Deer Due to Diet Overlap.” Animals 13: 561. 10.3390/ani13040561. 36830347 PMC9951756

[ece370529-bib-0070] Yang, C. T. , Y. Zhang , F. J. Hou , J. P. Millner , Z. F. Wang , and S. H. Chang . 2019. “Grazing Activity Increases Decomposition of Yak Dung and Litter in an Alpine Meadow on the Qinghai‐Tibet Plateau.” Plant and Soil 444: 239–250. 10.1007/s11104-019-04272-x.

[ece370529-bib-0071] Yang, K. , and C. H. Wang . 2019. “Water Storage Effect of Soil Freeze‐Thaw Process and Its Impacts on Soil Hydro‐Thermal Regime Variations.” Agricultural and Forest Meteorology 265: 280–294. 10.1016/j.agrformet.2018.11.011.

[ece370529-bib-0072] Yang, K. , J. J. Zhu , W. W. Zhang , et al. 2022. “Litter Decomposition and Nutrient Release From Monospecific and Mixed Litters: Comparisons of Litter Quality, Fauna and Decomposition Site Effects.” Journal of Ecology 110: 1673–1686. 10.1111/1365-2745.13902.

[ece370529-bib-0073] Yang, W. , W. Liu , A. Viña , et al. 2013. “Performance and Prospects of Payments for Ecosystem Services Programs: Evidence From China.” Journal of Environmental Management 127: 86–95. 10.1016/j.jenvman.2013.04.019. 23685121

[ece370529-bib-0074] Zhang, J. F. , J. G. Zhou , H. Lambers , et al. 2022. “Nitrogen and Phosphorus Addition Exerted Different Influences on Litter and Soil Carbon Release in a Tropical Forest.” Science of the Total Environment 832: 155049. 10.1016/j.scitotenv.2022.155049. 35390393

[ece370529-bib-0075] Zhang, M. H. , X. L. Cheng , Q. H. Geng , Z. Shi , Y. Q. Luo , and X. Xu . 2019. “Leaf Litter Traits Predominantly Control Litter Decomposition in Streams Worldwide.” Global Ecology and Biogeography 28: 1469–1486. 10.1111/geb.12966.

