# Supplementary material for: Wild Ungulates and Cattle Have Different Effects on Litter Decomposition as Revealed by Fecal Addition in a Northeast Asian Temperate Forest
Source: Ecol Evol. 2024 Nov 14;14(11):e70529. doi: 10.1002/ece3.70529 (PMC11563707; doi:10.1002/ece3.70529)
Supplement: Supplementary file 1 — Appendix S1‐S4. [file ECE3-14-e70529-s001.docx]

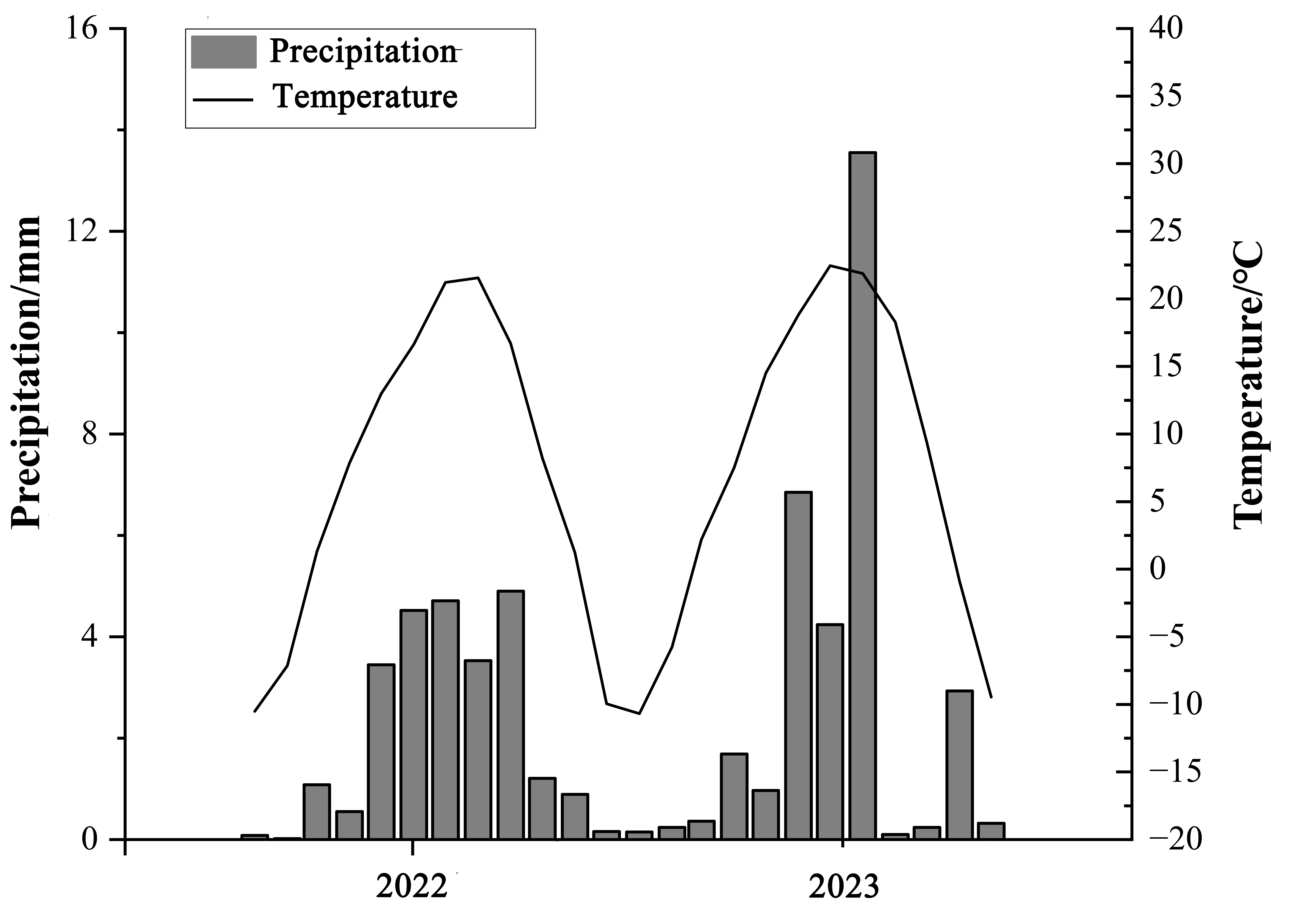


**Appendix S1** Monthly average air temperature and monthly precipitation. The study selected temperature and precipitation data from the Daily meteorological dataset of basic meteorological elements of China National Surface Weather Station (V3.0), and collected temperature and precipitation data from the Jilin Hunchun Meteorological Station (No. 54291) from July 2022 to October 2023.


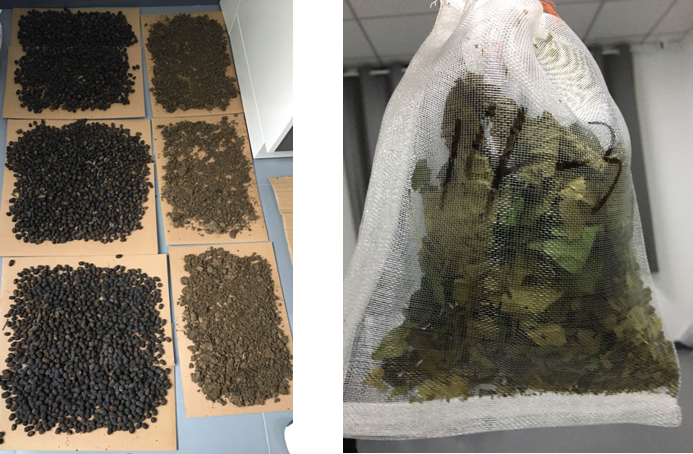


**Appendix S2** The left picture shows sika deer feces (left) and cattle feces (right) collected in the field, and the right picture shows the litterbag.


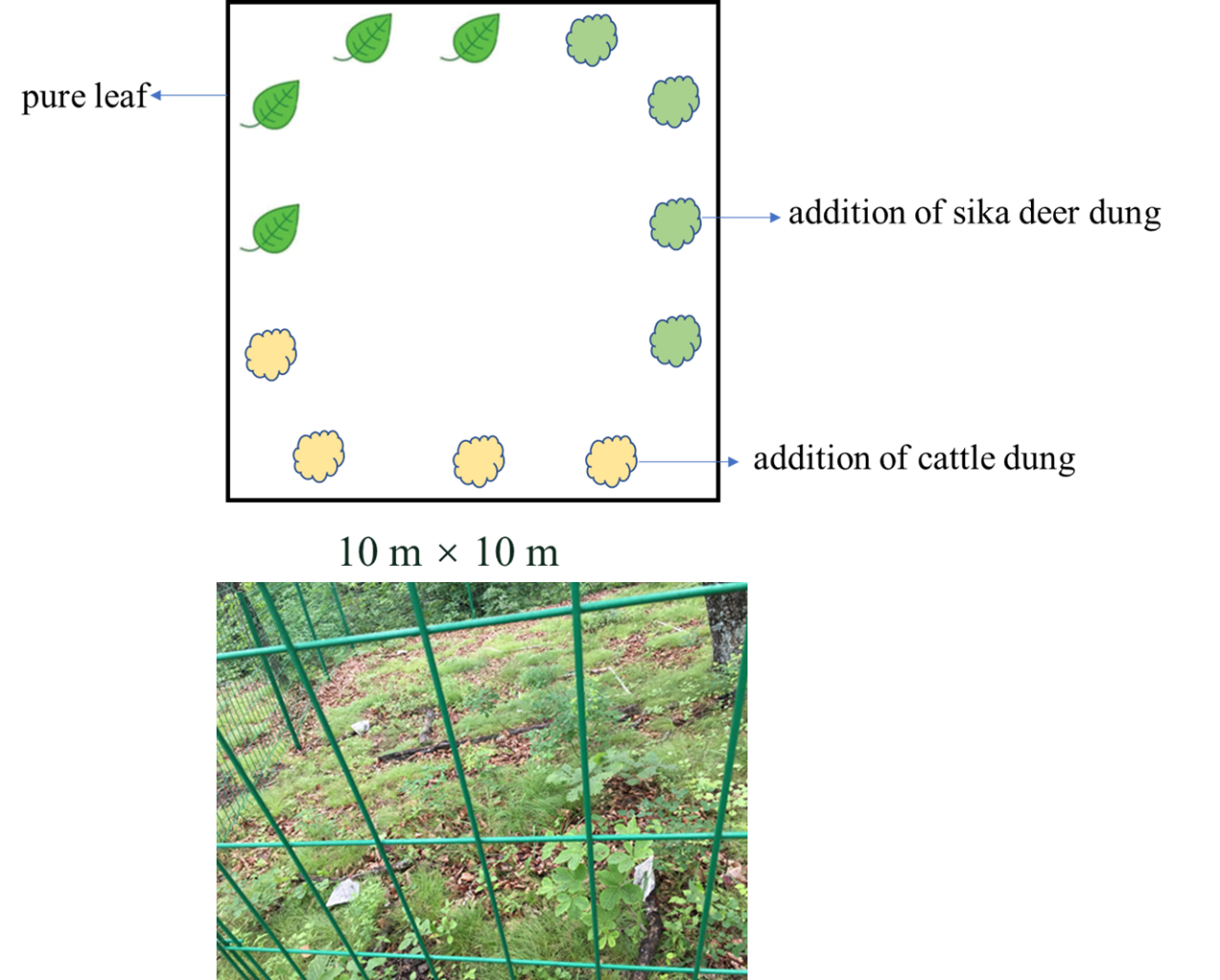


**Appendix S3** Schematic diagram of litter decomposition litterbag placed in the field.


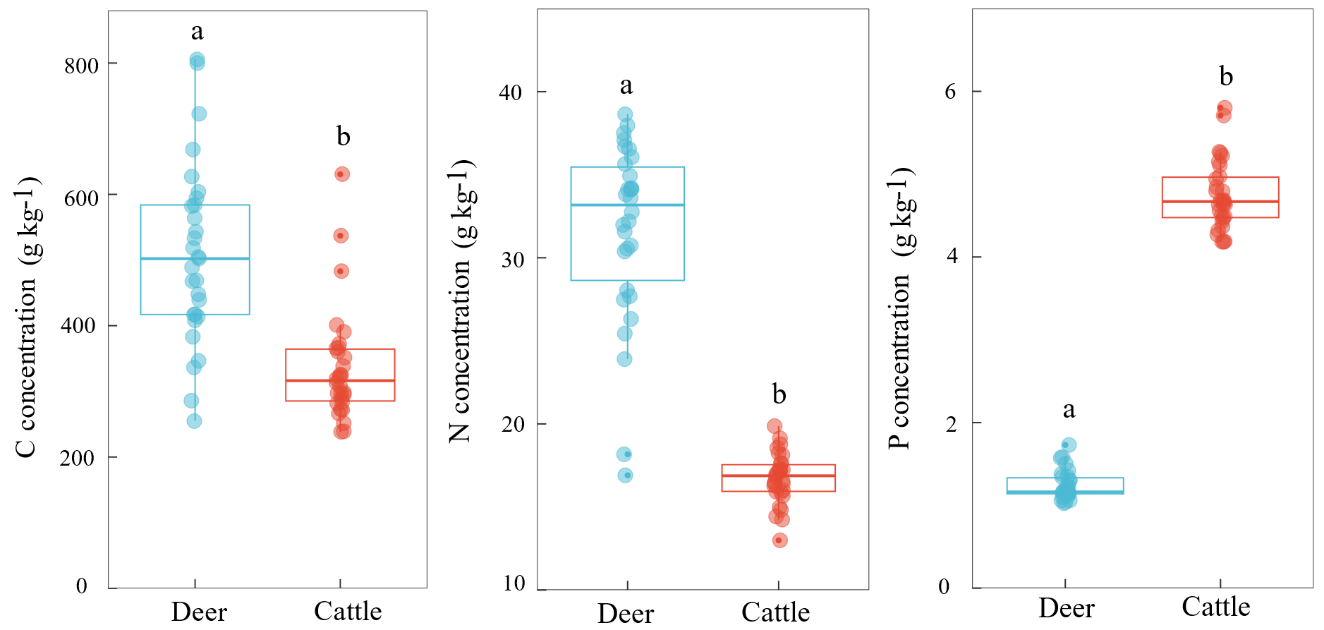


**Appendix S4** Comparison of initial C, N and P concentrations in feces between sika deer and cattle. Different letters within columns indicate significant differences (*P* < 0.001).
